# Effectiveness of an inactivated paratuberculosis vaccine in Iranian sheep flocks using the *Mycobacterium avium* subsp *paratuberculosis* 316F strain

**Published:** 2018-04

**Authors:** Rouholah Keshavarz, Nader Mosavari, Keyvan Tadayon, Masoud Haghkhah

**Affiliations:** 1 Department of Pathobiology, School of Veterinary Medicine, Shiraz University, Shiraz, Iran; 2 Razi Vaccine & Serum Research Institute, Agricultural Research, Education and Extension Organization (AREEO), Karaj, Iran

**Keywords:** *Mycobacterium avium* subspecies *paratuberculosis*, John’s disease, IS*900*, F57, Vaccine

## Abstract

**Background and Objectives:**

Paratuberculosis (PTb) (John’s disease) is an incurable chronic intestinal infection that mainly affects ruminants. PTb is caused by *Mycobacterium avium* subspecies *paratuberculosis* (MAP) with a global distribution. Despite evidences on MAP contribution in Crohn’s disease its causal role is still a matter of controversy. In ruminant farming, vaccination is broadly accepted as an effective control measure of PTb. This article describes preparation and field trial of an inactivated PTb vaccine made from the MAP 316F strain.

**Materials and Methods:**

Formulation of the vaccine was conducted based on the method traditionally used in the UK. Identity of the MAP strain was authenticated by PCR-IS*900* and PCR-F57 tests. In the field, a group of 100 lambs (3–8 weeks old) were subcutaneously inoculated with the vaccine preparation under study. These animals, pre-vaccination, were all PTb ELISA negative. Serum level of antibody was determined by ELISA on days 0, 30, 60, 120 and 240, post-vaccination.

**Results:**

In PCR-*900* and PCR-F57, the MAP 316F strain produced two fragments of 560 and 704 bp length respectively, a confirmation of its identity as MAP bacterium. In the field trial and at the arranged time intervals, the achieved blood serum levels of antibody, attributable to the vaccine formulation, displayed considerably high values.

**Conclusion:**

Given that the PTb-caused economical losses in the Iranian environment are dramatically high and also the fact that future of state policy on control of PTb remains unknown, we belive vaccination of animals is the best recommendable practice.

## INTRODUCTION

Paratuberculosis is an insidious, debilitating and very often serious enteric disease of ruminant populations across the world. Infection of farms with *Mycobacterium avium* subspecies *paratuberculosis* (MAP) the long-known single cause of the disease, leads to drastic losses due to interference in animal trading, reduced productivity and mortality on farms. No single strategy is available to eradicate the disease since the conventional diagnostic methods for tracking sub-clinically infected hosts are not error-proof (1). Improved management and hygiene measures, test-and-slaughter schemes to trace and cull MAP serologic/fecal-positive animals along with vaccination are the three globally accepted strategies in PTb control. Even in the developed world where lack of funding is not a constraint, control of the disease using the first two mechanisms is achieved very slowly with the condition of continuous efforts and dedication from farmers (2).

The earliest available report on PTb vaccination comes from the work by Vallée and Rinjard in 1926 (3). There is no written report on PTb vaccination until 1960 in sheep (4) and 1985 in goat (5). Worries are in place with safety and efficacy of currently available vaccines and potential interference of PTb vaccination with bovine tuberculosis tests due to cross-reactivity between MAP antibodies and bovine tuberculosis (BTb) antigens (2). Vaccination of farm animals however, has received increasing acceptance as an effective management strategy in control of PTb (6). Therefore, new ideas for designing and development of various novel vaccines are fast emerging (1, 7). In small ruminants PTb vaccination experience is less restricted as these animals are not normally included in tuberculosis eradication programs. While the therapeutic effect of PTb vaccination in small farm animals is less understood, administration of vaccine is recommended as early as the first months after birth since protection is expected to develop before the first contact of the animal with the pathogen (8). The original vaccination work by Valee and Rinjard was followed by numerous whole-cell, live attenuated and inactivated vaccines formulated for use in bovines and ovines (1). Gudair^®^, Silirum^®^, Mycopar^®^ and Neoparasec^®^ are the four commercially produced PTb vaccines with Mycopar^®^ (Boehringer Ingelheim Vetmedica Inc.) the only one licensed for use in the US cattle industry (3). This is a bacterin preparation derived from *M. avium* subsp. *avium* strain 18 which renders the vaccine not ideal as it lacks optimal antigenic repertoire (9). All the other three bacterin vaccines are prepared from the MAP strain 316F where Silirum^®^ (heat-killed, Zoetis Animal Health), Gudair^®^ (heat-killed, Zoetis Animal Health) and Neoparasec^®^ (live-attenuated, Rhone-Merieux) are approved for use in cattle, sheep/goat and sheep/goat, respectively (9).

The first PTb cases in farm animals of Iran surfaced in early 1960s with importation of exotic European breeds of cattle, sheep and goats blamed for introduction of the disease to the country (10, 11). PTb, however, is now a frequently reported disease of farm ruminants in Iran but is still not included in the state-funded disease control schemes. In 1970s, Hedayati and co-workers conducted experiments on PTb vaccination of sheep in Shiraz and reported success in induction of protection in flocks constantly suffering from the disease (Hedayati, unpublished material). He used the British Wey-bridge-developed procedure for preparation of the heat-killed whole-cell vaccine. In 1990s, a second experiment using the same methodology resulted in removing PTb from a government sheep flock in Kordan, a suburb of Karaj (Mosavari, Tadayon and Shahmoradi, unpublished material).

The present work focuses on laboratory preparation and field trial of an inactivated bacterin PTb vaccine made from the MAP 316F strain.

## MATERIALS AND METHODS

### Mass bacterial culture and genome extraction.

In order to obtain enough bacterial mass, five glass Fernbach flasks containing 1 L of Dorset-Henlley medium with no mycobactin J supplement were inoculated with the MAP 316F sub-strain and incubated for 12 weeks until expected mass bacterial growth was obtained.

### Genomic experiments.

Genomic material was extracted as described elsewhere (12). For authentication of strain identity IS*900* and F57 genetic markers were employed. In PCR-IS*900* and PCR-F57, Dohman’s and Schonenbrucher’s methods were used respectively, with brief modifications (13, 14). For PCR-IS*900* primers MAP-IS*900*f (5′ TTCTTG-AAG-GGT-GTT-CGG-GGC-C 3′) and MAPIS900r (5′ GCG-ATG-ATC-GCA-GCG-TCT-TTG-G 3′) and for PCR-F57 primers MAP-F57f (5′ ACCGAA-TGT-TGT-TGT-CAC-CG 3′) and MAP-F57r (5′ GGA-CAC-CGA-AGC-ACA-CTC-TC 3′) were used. The 15 μl reactions consisted of 7.5 μl of a commercial master mix (Ampliquor^®^, Denmark), 0.5 ML of each of the forward and reverse primers, 4 μl DNA template plus 2.5 μl DD water to fill up the remaining volume. Double distilled PCR water was used as negative control. Amplification cycles included an initial denaturation at 95°C for 5 min. followed by 35 amplification cycles of 30 s at 95°C, 30 s at 61°C and 45 s at 72°C with a final extension treatment at 72°C lasting for 10 min.

### Vaccine preparation: Inactivation of bacteria.

In a 50 ml Falcon tube bacterial mass transferred from a full-grown culture flask was submerged with 35% formaldehyde (One ml of formaldehyde per one gram of the wet bacteial growth) overnight to inactivate the mycobacterium. To ensure completion of the inactivation process, the Falcon tube was transferred to a pre-warmed waterbath and remained heated at 72°C for 2 hours. This was followed by centrifuge of the tube content (4,000 g/20 min). The supernatant was removed and the remaining pellet was hot air-dried (60°C) overnight. Using a pestle and mortar the dried pellet was grinded into a fine powder.

### Vaccine formulation.

Two grams of pumice powder was added to 150 mg of the fine powder from previous stage. One hundred and fifty milliliters of liquid paraffin plus 150 ml of olive oil were added to these two ingredients and the whole mixture was blended into a smooth paste preparation that was then stored at 4–8°C until its administration as vaccine.

### Quality control tests: Sterility.

In accordance with the recommendations made by WHO, 300 μl of the prepared vaccine was used to inoculate each of the two Thio-glycollate and Trypticase Soy Broth (TSB) tubes. The test was conducted in duplicates. Incubation and observation of the inoculated tubes (both 25 and 37°C) lasted fortnightly in search for traces of turbidity and precipitation.

### Safety.

Five hundred microliters of vaccine was administrated subcutaneously to two male guinea pigs (300–500 g). Inoculated animals were housed in a well-ventilated and comfortably warm place and any change in their body weight, necrosis at the injection site and development of local/general condition was monitored for eight weeks when all the animals were eventually euthenized and subjected to comprehensive post mortem examination.

### Potency.

Nine male guinea pigs (400–600 g) with no previous record of exposure to MAP were selected. To prepare the antigen suspension required for sentitization of animals, 100 ml of sterile paraffin was added to 400 mg of fine bacterial powder and 800 mg of pumice powder to make a smooth paste-like preparation of sensitizing material. All animals received their sensitizing injections (0.5 ml) through deep interamascular inoculation in their right thiges. Thirty days post sensitization, flank skin hair of the sensitized guinea-pigs were chemically removed and using a 2 × 3 latin square design, 200 μl of three dilutions (1:100, 1:500 and 1:2500) of vaccine preparation along with three dilutions of Avian standard PPD tuberculin (Avian PPDs) (1:100, 1:500 and 1:2500) were administrated intradermally in the flanks of these aniamls. Reading of the results was made 24 h post-injection when using a precistion caliper the two largest observable diameters of the readiness zone around injection sites were measured. The average of the two readings for any given site was calculated and recorded as the final reading.

### Immunization and duration of immunity.

Seven hundred lambs (3–8weeks old) from a sheep flock in Bojnord, North Khorasan province, with a history of ongoing PTb were subjected to a predesigned absorption ELISA screening test in search for detectable serum levels of antibodies against MAP. Subsequently, 200 animals with negative readings were selected and separated in two equal groups (100 animals, each) of vaccine and control animals. In the vaccine group, one ml of the prepared vaccine was injected subcutaneously in the right side of animals brisket region. Lambs in the control group received no injection. The likely changes in serum level of antibody in both groups of animals were observed through a domestically-developed ELISA system on blood samples taken on days 0, 30, 60, 120 and 240.

### ElISA assay.

Development of this in-house ELISA system was described elsewhere. In brief, culture filtrate (CF) antigens were coated to standard ELISA micro plates using bicarbonate buffer at 0.1 M (pH: 9.6). The coated micro plates were incubated for 18 h at 4°C. This was followed by a triple whashing treatment with 10mM PBS. Further coating of plates was blocked through filling the wells with 150 μl of 2.5% BSA (Sigma-Germany) and the whole preparation process was completed by incubation of plates for one hour at room temperature. The 10 mM PBS/Tween 20 and 1% BSA buffers were used to prepare 1/100 dilutions of serum samples.

In order to increase specificity of the ELISA system with no interfering impact on its sensitivity an extra preadsorption step employing *Mycobacterium phlei* was perfortmed. This was conducted through a 30 min-long preincubation of serums with 50 mg/ml solution of dried powder of *M. phlei* at room temperature. One hundred μl of treated serum was transferred to a fresh coated ELISA plate. This was followed by incubation at room temperature for 30 minutes when the plate was rinsed for 5 times with washing buffer. The next step was to add 100 μL of Horse Radish Peroxidase (HRP) conjugate (donkey anti sheep, abcam-UK) diluted by 10 mM PBS, pH 7.2) to the wells and incubation of plates for 30 min at room temperature followed by a washing phase. Subsequently, 100 μl of Tetra-Methyl Benzidine (TMB) substrate was added to wells and the plate maintained for 15 min at room temperature while protected from light. This was followed by adding 100 μl of a stop solution (HCl, 1 N) to the wells to finilaze the whole process when readings were made and recorded by an ELISA reader (BioRad-Model 620) at 450 nm.

## RESULTS

### Molecular observations.

In PCR-IS*900* and PCR-F57 amplification of MAP 316F genome, two amplicons of 560 bp and 704 bp were detected respectively- an indication of its identity as a MAP strain.

### QC tests.

No microbial growth of any kind was detected in the sterility test. Similarly no unusual reaction was observed during or at the end of the safety test as defined by most recent WHO guidelines.

### Potency test.

In the potency test all guinea pigs produced erythema reactions ranging between 8–25 mm. The average redness readings at 1:100, 1:500 and 1:2500 dilutions cosrresponding to vaccine preparation and standard PPD avian tuberculin were at 20.2, 14.7, 10.4 and 20.3, 15.9, 11.1 respectively (Table 1).

**Table 1:** Details of potency test readings

**Guinea pig ID**	**Average of erythema readings (mm)**					

	**Vaccine preparation**			**Standard**	**PPD**	**Avian**

	**1/100**	**1/500**	**1/2500**	**1/100**	**1/500**	**1/2500**
1	19	15	11	20	16	10
2	22	16	10	20	16	11
3	21	15	9.5	20	17	11
4	18	13	11	19	19	12
5	18	15	11	20	17	12
6	24	19	13	21	16	12
7	21	15	10	20	16	11
8	19	13	10	21	14	11
9	20	11	8	22	12	10
Mean (mm)	20.2	14.7	10.4	20.3	15.9	11.1

### ELISA.

Readings revealed a characteristic peak on day 30 in the test group which was followed by a continuous gentle rise in serum towards day 240. The OD values for days 0. 30, 60, 120 and 240 were 0.15, 1.6, 1.7, 1.8 and 2.1 for the test and 0.15, 0.2, 0.18, 0.18 and 0.2 for the control groups.

## DISCUSSION

Early generations of PTb ELISAs experienced a serious failure in their specificity due to cross reactions with environmental mycobacteria (15). This technical drawback was efficiently addressed through pre-treatment of serum specimens with a suspension of an environmental mycobacterium, *M. phlei* with no negative impact on sensitivity of the assay (15). Accordingly, in the present work an extra preadsorption step using *M. phlei* was conducted.

All currently available PTb vaccines suffer from a characteristic drawback so-called DIVA (Differntiating Infected from Vaccinated) as their application leads to an erroneous serological diagnosis of bTb infections (3). Fearing interference with the national test-and-slaughter plan against bovine tuberculosis (bTb), Iran has long been running a zero-tolerance policy against PTb vaccination in its cattle herd. Unless new generations of OIE-licensed PTb vaccine types bearing DIVA effect come to market, this strategy seems unlikely to change. On the other hand, Iran holds a considerably large flock of sheep as large as 48 million heads in 2017 (WAHIS Interface). This poulation is not covered by the national test-and-slauther scheme. Given the satisfying out-come from studies conducted in the rest of the world and the two previous field experiments by Hedayati and Mosavari together with observations from this study, one might assume vaccination of sheep flocks can be a very successful PTb control strategy in the Iranian environment.

Almost all commercially available PTb vaccines are based on MAP strains that are infact sub-strains of few classic strains collected early in the last century (16). Moreover, there are evidences to suggest that with reference to inactivated vaccine preparations, freshly-isolated local strains of MAP may be more effective (17). We believe, in any likely PTb vaccination plan of ruminants in Iran, application of indigenous MAP strains merits investigation.

Most of the commercial and non-commercial PTb vaccines use a trivial formulation including mycobacteria and a water-in-oil emulsion (liquid paraffin, olive, mineral, etc). A basic immunologic principle of all these vaccines is to enhance the immunogenesity of the preparation (3). Application of olive oil, liquid paraffin and pumice powder in the present study, therefore is justifiable though this is not a frequent property with most traditional veterinary vaccines.

Given the importance of economic objectives, development of a granulomatous lesion at the injection site, a typical drawback of oil-based bacterin vaccines (18), was not addressed here but remains for future works.
